# Linear Leucoderma Following Intralesional Steroid Injection

**DOI:** 10.7759/cureus.60783

**Published:** 2024-05-21

**Authors:** Anjana Beena, Ranjeeta Sapam, Gurumayum Chitralekha Devi, Anas LNU, Julie Leishangthem

**Affiliations:** 1 Dermatology, Jawaharlal Nehru Institute of Medical Sciences, Imphal, IND; 2 Dermatology, Venereology, & Leprosy, Jawaharlal Nehru Institute of Medical Sciences, Imphal, IND

**Keywords:** intralesional steroid injection, hypopigmented macules, depimented macules, leucoderma, linear leucoderma

## Abstract

Intralesional steroid injections avoid potential side effects associated with systemic administration, such as hypothalamus-pituitary-adrenal axis suppression, endocrine changes, allergic reactions, syncope, and blindness, but do not spare local side effects, such as pain, hemorrhage, ulceration, atrophy, hypopigmentation, calcification, secondary infection, granuloma formation, and allergic reaction. Linear leukoderma following intralesional steroid is a rare but known complication. Here, we report a case of a 23-year-old female presented with cutaneous linear depigmentation along the volar aspect of her left forearm developed three months following a single episode of injection triamcinolone acetonide for ganglion cyst.

## Introduction

In dermatology and beyond, intralesional steroid injections serve as a prevalent technique for administering corticosteroids directly to affected areas or lesions [1]. This method is highly regarded for its anti-inflammatory and immunosuppressive qualities, making it a widely adopted approach [1]. Intralesional steroid injections are commonly employed to address various medical conditions across multiple specialties. These include dermatological concerns, such as acne, alopecia areata, hypertrophic scars, and keloids. In addition, they are utilized in rheumatologic, neurologic (especially for multiple sclerosis), ophthalmologic (for periocular capillary hemangiomas and chalazions), osteoarthritis, and ganglion cysts [2]. Intralesional steroid injections offer a targeted approach, delivering potent doses of medication directly to the affected site and minimizing systemic side effects like hypothalamic-pituitary-adrenal axis suppression, endocrine changes, allergic reactions, syncope, and potential vision impairment [3]. However, local reactions can include discomfort, bleeding, ulceration, tissue atrophy, hypopigmentation, calcification, secondary infection, granuloma formation, and allergic responses [3]. Although rare, linear leukoderma can occur following intralesional steroid treatment [4]. This condition arises due to the depigmenting effects of steroids on the skin, resulting in linear patches of depigmentation aligned with the injection site.

Here, we report a case of a 23-year-old female presented with cutaneous linear depigmentation along the volar aspect of her left forearm developed three months following a single episode of injection triamcinolone acetonide for ganglion cyst.

## Case presentation

A 23-year-old female complained of a linear depigmented patch along the volar aspect of her left forearm. There was a history of a single injection of triamcinolone acetonide 40 mg/ml following aspiration for the treatment of a cystic lesion over the left wrist three months back. Her prior medical documents suggested the swelling as a ganglion cyst. One month following this, the patient noticed a depigmented patch over the site of injection. In two months, the patch extended from the wrist up to the elbow joint along the inner aspect of the forearm. It was not associated with itching or pain over the patches. Past and family history are non-contributory.

**Figure 1:**
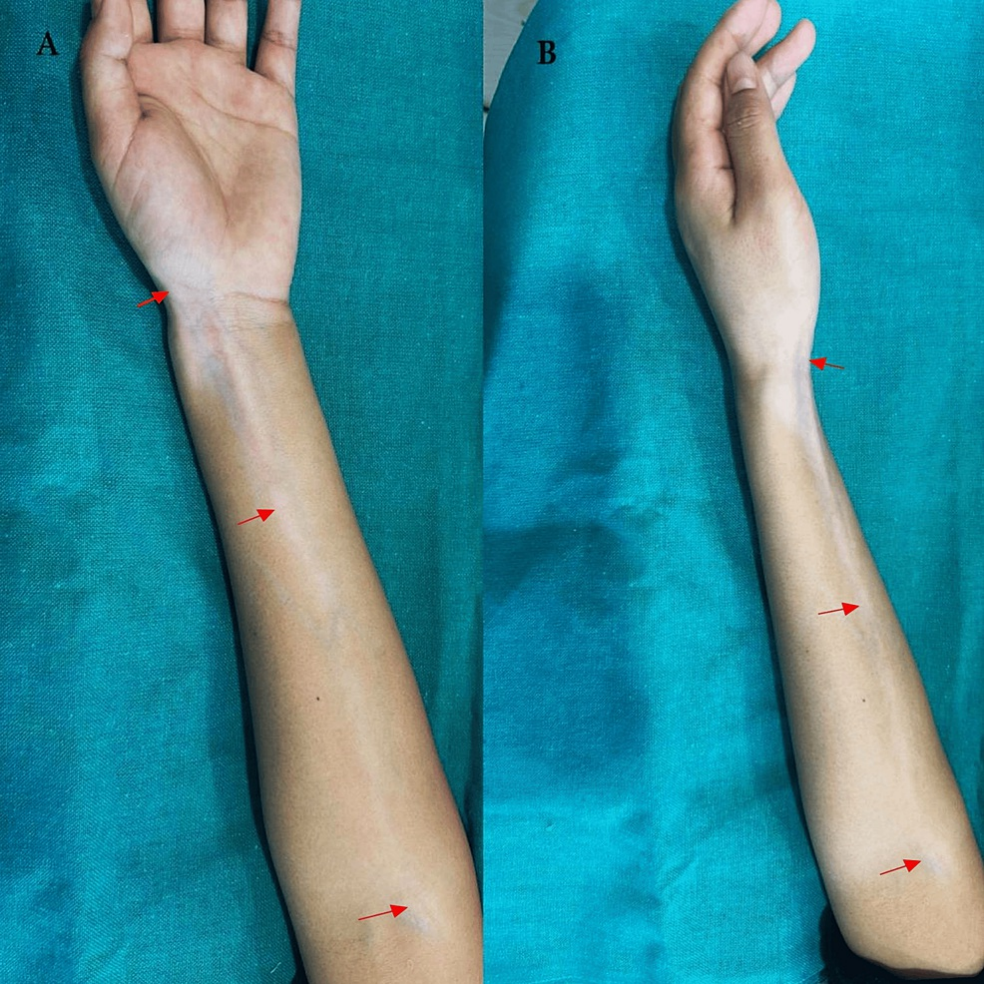
Linear depigmented macule (red arrow) extending from the wrist to the cubital fossa

On cutaneous examination, a linear depigmented macule of size 16 x 1.5 cm extended from the wrist along the medial side of the volar aspect of the forearm up to the cubital fossa (Figure 1A, 1B). No similar lesions anywhere else in the body.

**Figure 2:**
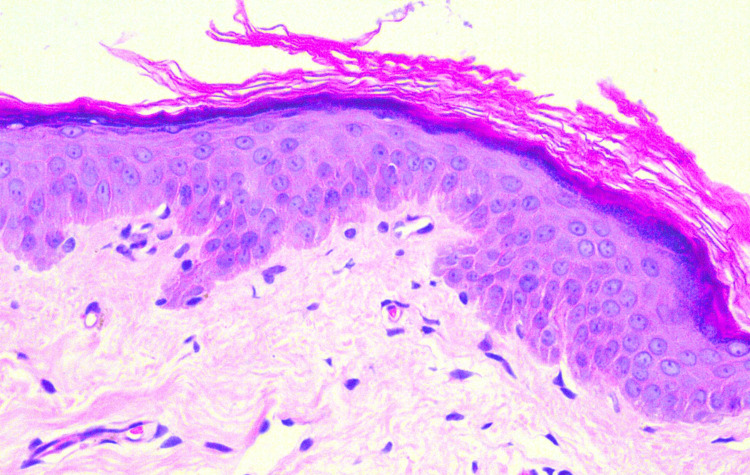
Hematoxylin and eosin stain The epidermis shows subtle papillomatosis with a normal number of melanocytes but decrease melanin content.

Biopsy for histological examination from the depigmented macule showed the epidermis displaying subtle papillomatosis with a normal number of melanocytes but decreased melanin content. The dermis and subcutaneous are unremarkable (Figure 2). Hence, a diagnosis of linear leukoderma following the intralesional injection of triamcinolone was made. The patient was counseled regarding the benign course of the condition, started on topical tacrolimus 0.1% cream for local application, and is under follow-up.

## Discussion

Although intralesional injections are an effective treatment modality, common local side effects include perilesional hypopigmentation, depigmentation, cutaneous atrophy, alopecia, infection, ulceration, and localized dystrophic calcification [2]. Topical steroids can lead to various cutaneous adverse events, such as sensations of stinging and burning, worsening of acne, delayed wound healing, dyspigmentation, thinning of the epidermis, hypertrichosis, skin atrophy, and increased vulnerability to bacterial, fungal, and viral skin infections [5].

Leukoderma, also referred to as achromoderma, is identified by distinct areas of depigmented patches resulting from the depletion of melanin in the epidermis. This condition stands apart from hypopigmentation, which entails a partial reduction in pigmentation rather than a complete absence [6]. Linear leukoderma, marked by a linear pattern of hypopigmented patches, is an infrequent complication following intralesional steroid injections, with limited documentation in the literature [4]. The precise mechanism behind corticosteroid-induced leukoderma remains uncertain. Proposed explanations include the inhibition of melanocytes through the suppression of enzymes vital for melanin synthesis, like tyrosinase, possibly leading to melanocyte depletion [7]. In addition, corticosteroids may suppress inflammatory responses by reducing the production of prostaglandins or cytokines in epidermal cells, thus potentially impeding the secretion of melanocyte metabolic products. Another suggested mechanism involves alterations in skin vasculature [8]. While the exact mechanism behind linear leukoderma remains elusive, it is hypothesized to stem from the presence of insoluble corticosteroid crystals within lymphatic vessels. This presence potentially initiates localized lipolysis, leading to skin depigmentation along the peri-lymphatic regions [4].

The degree of corticosteroid-induced hypopigmentation is influenced by both the concentration of steroids administered and the depth of injection (Table 1) [9]. At present, no specific treatment exists for this condition, and spontaneous repigmentation may take several months to manifest. Subsequent injections should be avoided. Initiating treatment with topical calcineurin inhibitors such as tacrolimus can expedite recovery in this patient [10]. In cases where initial treatment is ineffective, the fractional CO2 laser has shown promising results [11].

**Table 1:** Depth of injection of triamcinolone acetonide and its respective side effects.

Depth of injection	Side effect
Intra-keloidal (40 mg/ml)	Linear atrophic hypopigmention (Kaur et a.l)
de Quervain tendons (40 mg/ml)	Linear atrophic hypopigmention (Canturl et al.)
Intraganglion cyst (40 mg/ml)	Linear depigmentation (Dhawan et al.)
Intralesional into hypertrophic lichen planus (40 mg/ml)	Linear depigmentation (Dhawan et al.)
Intra-keloid (10 mg/ml)	Linear atrophic depigmentation (Madireddy et al.)
Intra-wrist joint (40 mg/ml)	Hypopigmentation of skin over the injected area (Gupta et al.)
Intra-ganglion (40 mg/ml)	Linear atrophic hypopigmention (Gunawat et al.)

## Conclusions

This case report highlights the importance of recognizing linear leukoderma, a relatively rare cutaneous adverse effect following intralesional steroids, which can cause cosmetic distress to the patient. Appropriate concentration and depth of injection can reduce this side effect, which practitioners should be aware of.
